# Three-Dimensional Printed Annular Ring Aperture-Fed Antenna for Telecommunication and Biomedical Applications

**DOI:** 10.3390/s24030949

**Published:** 2024-02-01

**Authors:** Khaled Alhassoon, Yaaqoub Malallah, Fahad N. Alsunaydih, Fahd Alsaleem

**Affiliations:** 1 Department of Electrical Engineering, College of Engineering, Qassim University, Unaizah 56452, Saudi Arabia; f.alsunaydih@qu.edu.sa (F.N.A.); f.alsaleem@qu.edu.sa (F.A.); 2 Energy and Building Research Center, Kuwait Institute for Scientific Research, Shuwaikh 70030, Kuwait; ymalallah@kisr.edu.kw

**Keywords:** annular ring antenna, aperture fed, 3D material, WBAN, 3D printing, IoT

## Abstract

The design of the aperture-fed annular ring (AFAR) microstrip antenna is presented. This proposed design will ease the fabrication and usability of the 3D-printed and solderless 2D materials. This antenna consists of three layers: the patch, the slot within the ground plane as the power transfer medium, and the microstrip line as the feeding. The parameters of the proposed design are investigated using the finite element method FEM to achieve the 50 Ω impedance with the maximum front-to-back ratio of the radiation pattern. This study was performed based on four steps, each investigating one parameter at a time. These parameters were evaluated based on an initial design and prototype. The optimized design of 3D AFAR attained S_11_ around 17 dB with a front-to-back ratio of more than 30 dB and a gain of around 3.3 dBi. This design eases the process of using a manufacturing process that involves 3D-printed and 2D metallic materials for antenna applications.

## 1. Introduction

The new communication and biomedical systems technology era demands adapting conventional RF circuits to meet the unique requirements [1,2]. Flexibility, tunability, miniaturization, and reliable feeding are the circuits’ most manufacturing-adapted and evolved features [3,4,5]. Each one of these features concerns investigating different parameters that relate to novel designs or materials. For example, the soldering of 2D materials is one of the biggest challenges with RF circuits [6] and requires an aperture-fed technique to ease the practicality of such new materials [4]. Moreover, 3D printing is a unique venue for RF circuits, promising especially novel antenna design for 5G telecommunications systems [7]. It can be employed to ease the implementation and realization process of a complex substrate of RF components [8,9,10,11,12,13,14,15,16,17]. However, some challenges, such as soldering, are associated with the 3D-printed substrate. The difficulty with the soldering process is that neither the 3D-printed substrate nor novel 2D materials can stand the high temperature of SMA connector soldering and will melt. As a result, an alternative feeding approach is required to have a solderless process. One of the alternative approaches is the aperture feed technique for microstrip antennas.

The annular ring antenna is attractive because of its high performance and small footprint compared to other antennas. It has the smallest area footprint, around half the area of other antennas, such as rectangular patch antennas [18]. This type of compact antenna can easily be included within an advanced system on a chip [19]. This is a crucial characteristic and is in demand for advanced telecommunication and biomedical applications. However, this type of antenna with an aperture-fed technique has not been introduced or investigated in the literature. Moreover, this designed aperture-fed annular ring antenna (AFARA) at 2.4 GHz is practical for use as a radiator with any newly developed 2D metal trace material. The solderless approach of aperture fed is practical since most of these materials have a limited capability to handle high temperatures and connect with other components. The contactless feeding method also eases the implementation process and provides more practicality. Finally, this feeding technique can reduce the cross-polarized effects, the surface wave, and the spurious radiation generated from the probe feeding technique [20].

The annular ring patch antenna is a disc antenna but with a circular gap in the center. Moreover, the resonant metallic radiator is smaller as there is a circular gap in the center [18]. As mentioned earlier, the structure contains a ring-shaped metallic resonator above the substrate, which can be fed by different techniques, and a ground plane underneath. There are three essential parameters: the outer radius (b), the inner radius (a), and the dielectric constant (c) [21]. These three parameters specify the resonant frequency and operational mode. In addition, the outer and inner radius ratio specifies the operating mode. 

The design aspects and specifications must be reconsidered to employ these new technologies in the RF field. The new 3D-printed technology and 2D metallic material open new venues to manufacture microwave components, specifically microstrip antennas [22,23]. The electrical characterization of the 3D-printed filament material needs to be improved. As a result, an investigation effort was conducted to overcome this issue [3,24,25]. Feeding is one of the most critical factors that impact performance and manufacturing [26]. Since the 3D-printed material of the substrate will melt at high temperatures because of soldering, the probe feeding must be substituted with aperture feed [1].

Moreover, 2D material such as MXene is delicate and cannot be soldered with a feeding circuit [27]. A study that resolved this challenge to characterize 2D solderless materials by introducing the capacitive coupling filter has been reported [6]. As a result, the aperture-fed method will exclude the need to solder the feed circuit and radiating elements of the antenna [28,29,30,31]. Most of the work related to aperture-fed antennas has been conducted on linearly polarized rectangular antennas [28,29,30,31,32,33]. However, the only paper that discusses the annular antenna is [34], but about circularly, not linear polarization. This paper focuses on the circular polarization annular ring antenna by exciting TM_11_ using L-shape feeding. Moreover, they changed the outer-to-inner radius ratio to 0.3 instead of 0.5. The aperture-fed technique of an annular ring antenna that is excited with linearly polarized TM_11_ has not yet been investigated in the literature. Even most of the reported research about 3D antennas did not study this type of antenna [23,26,35,36,37,38,39,40]. The proposed linearly polarized annular ring antenna consists of three layers and has not been designed nor investigated in the literature. This type of antenna will be attractive for wireless body area network (WBAN) applications and 3D-printing RF components [19,41,42,43]. This type of biomedical application that is used as a sensor requires a very limited bandwidth [44]. IoT and WLAN applications can also operate at 2.4GHz with limited bandwidth [45,46]. 

A paper by Mirzaee [47] reports that a 3D-printed dipole antenna is made using an ABS substrate and carbon paste as a conductive material. Similarly, the author used the same design with a different material, nylon filament [36]. Furthermore, one of the papers examines three different infill levels (40, 70, and 100% infill) for NinjaFlex filament. An antenna with a rectangular shape was printed with 100% infill. The gain of −4 dB is expected to be low due to the high loss tangent around 0.06. Based on the findings of [48], the author developed a hybrid substrate that combines NinjaFlex with ABS. Due to its flexibility and low loss, the 3D-printed substrate was highly effective. Approximately 5 dB of gain was obtained with the antenna [48]. Another paper outlined three different antenna designs after performing the PLA extraction: bowtie, spiral, and Yagi–Uda [49]. As in [50], fully printed antennas are presented, including the substrate, conductive material, and the SMA connector. Moreover, 3D printing of two orthogonal antennas was designed at 1GHz to reduce wireless communication interruptions [51]. The probe-feeding antennas are to combine electric and magnetic dipoles with a phase difference between them. Table 1 shows the reported antenna that was implemented using 3D filaments. All of the reported antennas used probe-feeding techniques.

All these papers are directly connected to the SMA using the soldering technique. Moreover, none of the conducted research succeeded in substituting the conventional copper sheet with a highly conductive material. As a result, the solution will be to introduce 2D conductive materials on the 3D aperture-fed antenna that is easily fabricated and excited with no damage. In this paper, by utilizing the solderless aperture-fed technique, the 3D substrate of the antenna will be intact, and no further loss will be introduced. This work lies in the comprehensive exploration and optimization of parameters, resulting in a highly efficient AFAR microstrip antenna. Moreover, there are significant improvements in the performance of using 3D substrates and ease of use with 2D materials. This study paved the way for considering the 3D and 2D materials for complex antenna implementation with contactless feeding.

This paper presents the parametric study, design, and implementation of the aperture-fed annular ring antenna. The simulation results of including 2D MXene antenna are designed and show the promising potential of using this design after we did the characterization of this material [6]. The final optimized practical antenna designs are presented, and measured performance is compared against modeling results.

## 2. Analysis

The aperture-fed annular ring antenna reduced the footprint by around 56% compared to the rectangular antenna and is designed mainly to examine the flexibility and practicality of using 2D material as a radiator. The solderless approach of aperture fed is practical since 2D materials such as the MXene layer have a limited capability to handle high temperatures [6,27]. Moreover, this feeding technique can reduce the cross-polarized effects, the surface wave, and the spurious radiation generated from the probe feeding technique [52].

The feeding line of the aperture-fed antenna creates an electric field in the aperture on the ground plane; This electric field induces a surface current on the patch. This aperture slot is a medium impedance transformer to couple the power from the feeding line to the patch. Thus, the substrate thickness of the feeding line is suggested to be thin to maximize the coupling power and reduce the potential radiation from the feeding layer. However, this approach will cause an unwanted, huge back lobe; because of that, an alternative design approach to minimize the back lobe will be discussed in the next section. The antenna substrate thickness prefers to be thicker to have a better gain. For example, the gain improved by around 3 dB when the thickness changed from 0.1 mm to 0.5 mm. 

### 2.1. Design 

The primary design of the aperture-fed annular ring antenna is based on Equation (1) [52].

(1) $$f_{nm}=\frac{\chi_{nm}c}{2a\pi \sqrt{\epsilon }}$$

where *c* is the velocity of light in free space, $\chi_{nm}$ is the Nth root of the Bessel equation of order m of the characteristic equation using Bessel, *ϵ* is the dielectric constant, and b/a = 2. The root of the Bessel function $\chi_{nm}$ is around 0.6. However, this is used for probe feeding the ring antenna at the inner edge. The probe feeding was excluded from designing the aperture-fed ring antenna. The complex permittivity of the materials used is listed in Table 2. The baseline antenna, which used RT/druid and PET materials, is considered the initial stage of the design. Further improvement and investigation were performed to design the optimized 3D-printed antenna. The dimensions of the 2.4 GHz aperture-fed annular ring are listed in Table 2.

**Figure 1:**
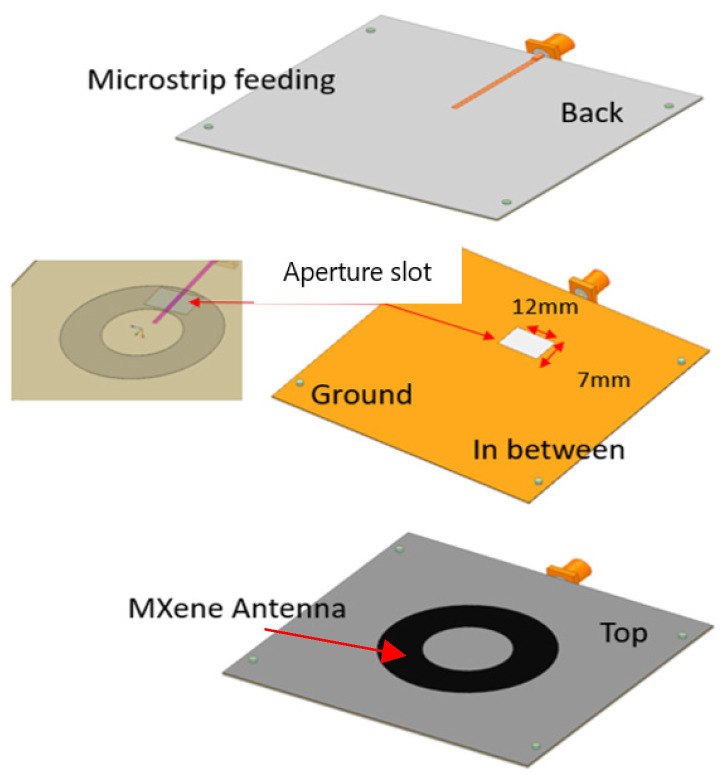
Aperture-fed annular ring antenna design with annotation showing the aperture feeding and the conductive material.

The proposed linearly polarized annular ring antenna has three layers, as shown in Figure 1. The back layer is the 50 ohm feeding line connected to the SMA connector, which is used instead of the probe feeding. Then, the ground plane underneath has an aperture to couple the RF signal to the radiator. The top layer includes the radiator, where the power is coupled to it to radiate.

The aperture slot position and dimensions were optimized using the finite element method HFSS to achieve optimum performance. The optimization criteria include good matching, maximum power transfer, operating mode TM_11_, and smaller back lobs. The position of the aperture is 0.08 λ_g_ (5.3 mm) from the end of the feeding line. Moreover, the wider the aperture, the better performance, but it will cause a large back lobe.

Furthermore, the increase in width or length of the aperture causes the resonant frequency to increase, the gain at the resonance to increase, the input resistance to decrease, and the input reactance to increase. After optimizing and evaluating the gain at various sizes, the final dimensions are 12 × 7 mm, which is 0.18 × 0.1 λ_g_. However, this initial design has a huge back lobe with a front-to-back ratio of about 10 dB. An additional investigation to reduce the back lobe is required to have a smaller back lobe.

The initial antenna design with PET substrate was perfect for matching and resonance frequency for baseline, but the radiation pattern’s front-to-back lobe ratio is less than 10 dB. The significant observation is that the feeding line tends to have larger back lobes than thicker ones on a very thin substrate. Therefore, modifying and further optimizing the design to have a maximum front-to-back ratio is very important. This issue can only be resolved by examining the source of this huge back lobe and finding an alternative solution. The initial design investigation concludes that the thin substrate of the feeding line is the primary source and requires the use of a thicker substrate, as shown in Table 3. Moreover, increasing the thickness of the feeding substrate layer will cause a mismatch, even if it reduces the back lobe, as shown in Table 3. One potential solution is reducing the width of the aperture and increasing the length to have a better match. As a result, a new design methodology will be followed to overcome all these issues.

### 2.2. Parametric Study

**Figure 2:**
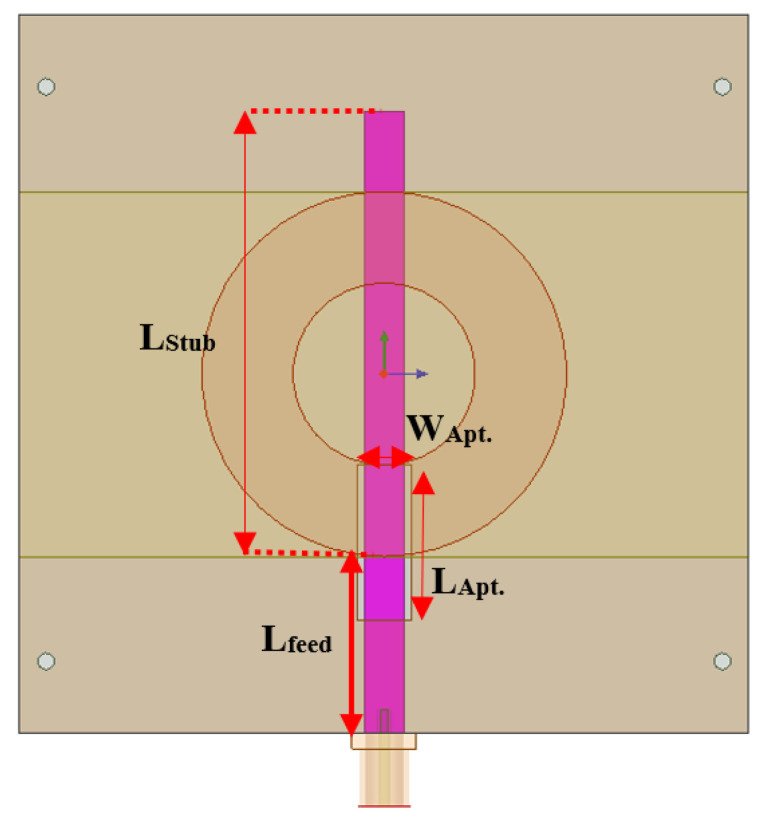
Aperture-fed annular ring antenna structures with annotation showing aperture slot and transmission line dimensions.

The new approach is inspired by a publication that deals with designing aperture-fed circularly polarized annular ring antenna [34]. In this section, the design of a 3D-printed substrate aperture-fed antenna is investigated. In this optimization-investigated study, four essential parameters must be analyzed using a numerical approach to enhance the design of a 3D-printed antenna. These are the feeding length of the 50 Ω feeding line (L_feed_), the length of the stub of the feeding line (L_Stub_), the width of the aperture on the ground plane (W_Apt._), and the length of the aperture (L_Apt._), as depicted in Figure 2. The two substrate layers of the antenna have the same thickness of 60 mils but different ABS filament infill. The top substrate layer where the annular ring is attached is printed with 50% infill. However, the back substrate layer, where the feed line is attached, is printed with 100% infill to have a wide 50 Ω feeding line. The reason for choosing low infill for the top layer is for a higher gain when having a low dielectric constant. The parametric study will follow four steps of investigation and optimization. The analysis is performed only on one parameter among the four at a time, and the others maintain the same. 

### 2.3. Investigation Steps

Step one investigates the appropriate length of the feeding line L_feed_. After sweeping different lengths using HFSS simulation, it is observed that the shorter the L_feed_, the lower the impedance and bad matching. However, the small impedance of the real part can be matched by adjusting the stub L_Stub_ length. The best length fits the design when the imaginary part is small. Thus, the chosen L_feed_ length is $\lambda_{g}/4$ (19.5 mm) of the feeding substrate. The next step is to study the stub length, which is the most important for better matching.

The next step is sweeping the L_Stub_, which extends from the end length of L_feed_, which is aligned with the edge of the outer radius of the annular ring in the upper layer. It is clear that the behavior is not following an obvious pattern. This uncertainty behavior is due to different interactions with the aperture where different lengths fall on the inductive or capacitive region of the Smith chart. However, as this design’s purpose is to reduce the aperture width, it primarily controls the real part impedance; thus, we need to choose the shortest length with a close impedance to 50 Ω. As a result, this will be 50 mm long, as tabulated in Table 4. This further optimization is required once we finalize the aperture dimensions. The total length of the 50 Ω feeding line width is around 0.87$\lambda_{g}$ (68 mm) which is the sum of L_feed_ and L_Stub_. This length of the feeding line will increase the total size of the substrate since the open end of the feedline cannot end at the edge of the substrate. 

The aperture location in the ground between the two layers is the transfer media between the feeding line and the excited radiator. The aperture has two components, width (W_Apt_) and length (L_Apt_), that govern the impedance matching and impact the radiation pattern. After the substrate thickness of the feeding line was changed from the initial design to the thicker, a mismatch appeared clearly from the return loss, but the back lobe was reduced by around double. This is shown in Table 5 for different aperture widths, and the length is fixed at 7 mm. Reducing the width will cause a mismatch and lower the antenna’s efficiency. The same sweeping was carried out for the aperture length, but the width was fixed to 12 mm. The higher the length, the better the matching, but it was still unacceptable, as shown in Table 6. A significant conclusion is that reducing the width and increasing the length of the aperture is the approach to reaching a good match antenna with a low back lobe.

Taking one step back is essential to choose the best width from Table 5. The smallest aperture width that can let the antenna resonate is desired to have a low back lobe for this design. This width is 6 mm, which has very poor matching but a reasonable gain and back lobe level. However, the mismatching can be improved by increasing the length of the aperture, as explained earlier. The width was set up to 6 mm, and swept the length, as shown in Table 7. As predicted, the more considerable length of L_Apt_ provides better matching but a lower back lobe with a consistent frequency. For example, the 26 mm length has a better return loss than 16 mm. Nevertheless, it does have a lower front-to-back ratio of the radiation pattern, which is 19 dB as opposed to 20 for the shorter length. This finding concluded that the aperture length is a trade of impedance matching and back lobe level.

**Figure 3:**
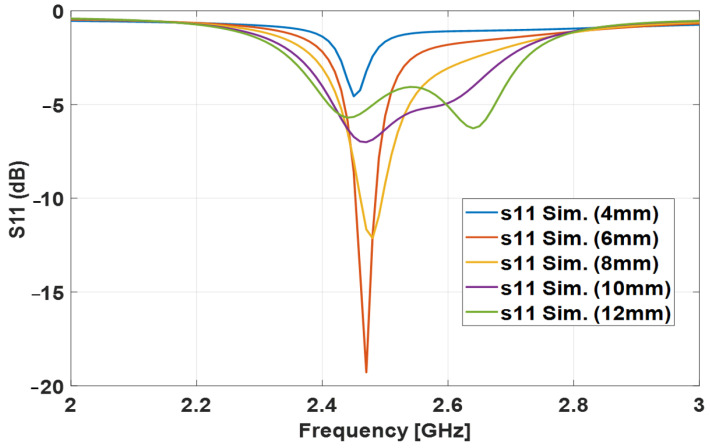
Simulated return loss of various sweeping aperture widths with fixed length (17 mm).

The final step is to confirm and optimize the design. This step was performed by sweeping the different aperture widths for the optimum length which is 17 mm. As seen clearly in Table 8, the 6 mm width has the best return loss and front-to-back ratio level. This is because, at these dimensions, the antenna is exposed to the least inductive, which increases the antenna efficiency. The return loss of the different widths is shown in Figure 3.

The optimized 3D (AFAR) antenna resonates at 2.4 GHz with S11 around 20 dB. The dimensions in terms of the guided wavelength of all antenna parameters are listed in Table 9.

The 2D material MXene will be used with the optimized design in this step. Instead of using a copper sheet of 5.96 × 10^7^ S/m conductivity for the annular ring radiator, a thin film of thickness 4.3 μm MXene (Ti_3_C_2_T*x*) of 1.2 × 10^6^ S/m will be implemented [6]. This step will introduce further conductive losses to the designed antenna. However, it will help to introduce and implement novel ideas for biomedical applications. The simulation results of resonant frequency is around 2.47 GHz shifted about 40MHz upward from the copper sheet antenna. The S_11_ dB is around 8 dB and this impedance mismatching is due to conductive loss introduced when employing the MXene. Additionally, the gain was reduced by 1 dB to be around 2 dB, and the front-to-back lobe was around 19 dB.

## 3. Fabrication

The aperture antenna designs of the 2.4 GHz dimensions are tabulated in Table 2. The first antenna is the baseline with PET layers, and the second is 3D printed. The two layers with common ground in between were printed using a fused filament fabrication (fff) 3D printer with ABS material. A characterization process was performed as a 3D filament material of the printed substrate, which is crucial for accurate design. This extraction process was based on curve fitting of least square error using an annular ring resonator and transmission line. These characterization processes follow the same work that was reported in [5], with the difference being using a Prusa 3D printer and other ABS filaments. The ABS material’s electromagnetic properties were extracted using a transmission line. The complex permittivity of 100% infill is 2.5–0.12i, and 50% infill is 2.2–0.09i.

**Figure 4:**
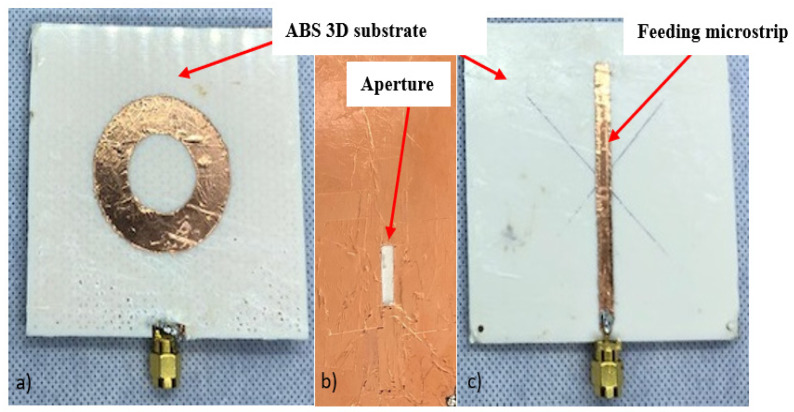
Aperture-fed annular ring antenna structures layers: (**a**) radiator on 50% ABS 3D-printed substrate; (**b**) ground plane with aperture; (**c**) feeding microstrip line on 100% ABS 3D printed substrate.

As there are two layers of common ground in between, as shown in Figure 4, the fabrication was performed in a couple of steps. Figure 4a is the first layer where the patch is attached to a 3D printer ABS substrate of 50% infill. Figure 4b,c depict one layer with the aperture milled in the ground plane at the top and a feeding line at the back side of the 3D printer ABS substrate of 100% infill. After the two layers were printed, the milling copper sheet was attached. Figure 4a is the aperture antenna realization after attaching the two layers using double-sided tape and is ready for testing. 

## 4. Measurement

**Figure 5:**
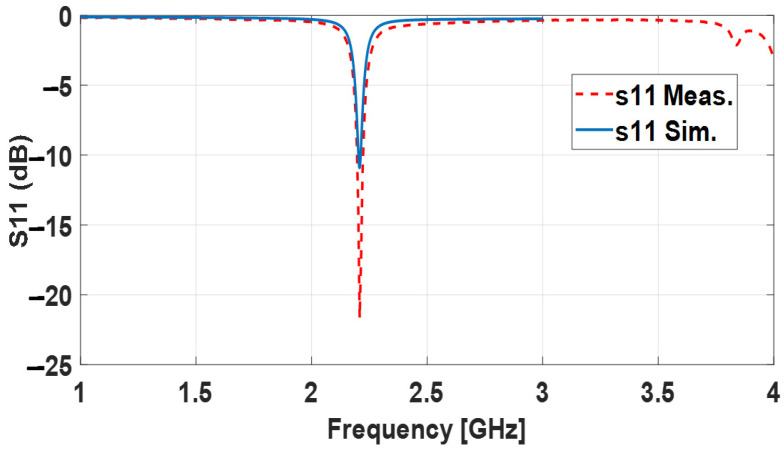
Measured and simulated S_11_ dB of 2.4 GHz baseline aperture-fed annular ring antenna.

In the initial baseline design, the aperture annular ring antenna resonates at 2.2 GHz with a good matching of around 22 dB. The measurement was performed using a Tektronix TTR506A USB Vector Network Analyzer BN533844 from Tektronix, Beaverton, Oregon, USA. Figure 5 shows the return loss of the measured and simulated baseline aperture-fed annular ring antenna. The simulation matches the measurement well as predicted. It is worth mentioning that the bandwidth of 30 MHz is larger than the probe feeding antenna of the same frequency. Even though the thickness of the aperture-fed patch’s substrate is 15 times less than the probe feeding, the bandwidth is twice as high. This is because the aperture antenna includes two resonances: the patch and slot. When these resonators are coupled well, the two resonances become close, leading to higher bandwidth [53].

**Figure 6:**
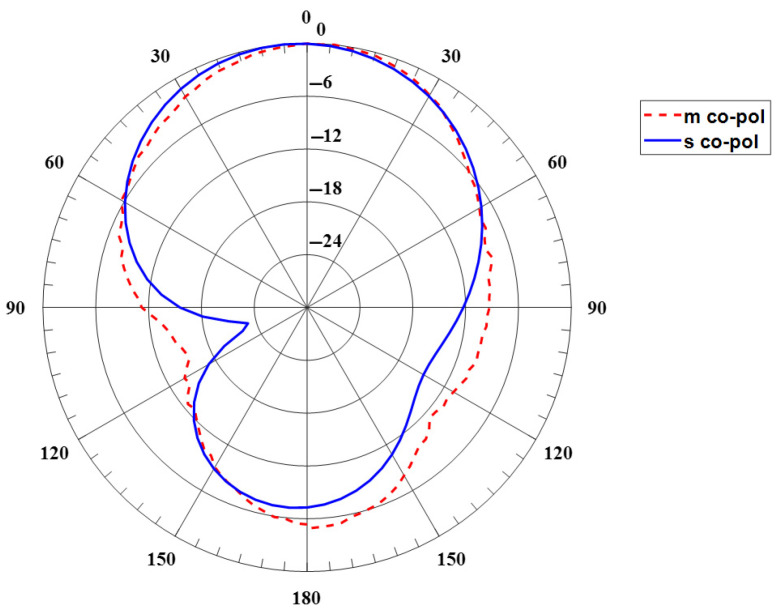
Simulated and measured the E-plane radiation pattern of the baseline annular ring antenna.

**Figure 7:**
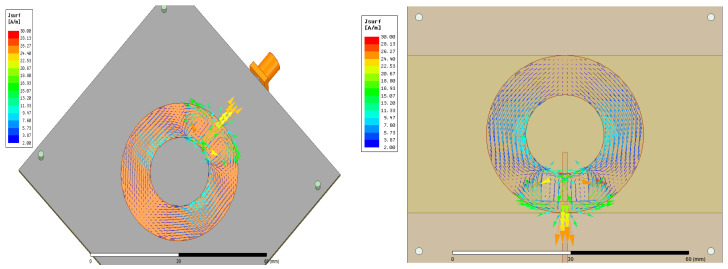
Simulated E&H-plane radiation pattern of baseline annular ring antenna and current distribution of TM_11_ mode.

Figure 6 shows the far-field radiation pattern. The measurement was performed in the Anechoic Chambers at 2.2 GHz. The pattern is radiated on the broadside and has a huge back lobe. Figure 6 also depicts the simulated E-plane radiation pattern. However, the gain is low around −5 dB since the patch’s substrate is thin. This can be enhanced by using a thicker substrate. Figure 7 depicts the surface current on the ring patch, which operates at TM_11_ mode.

**Figure 8:**
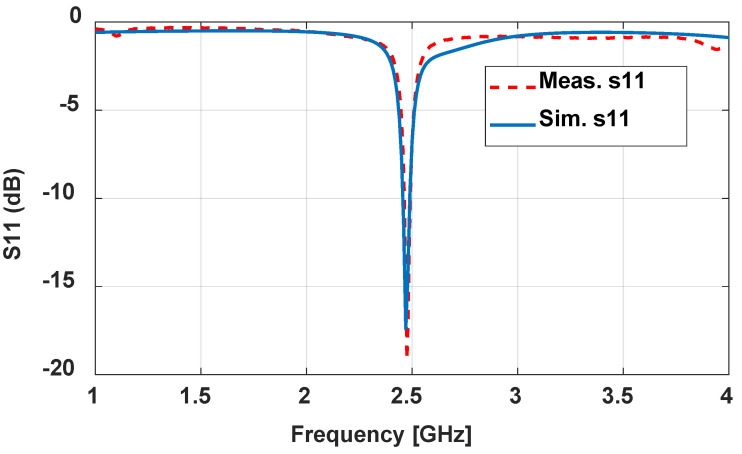
Measured and simulated return loss of 3D aperture-fed annular ring antenna at 2.4 GHz.

**Figure 9:**
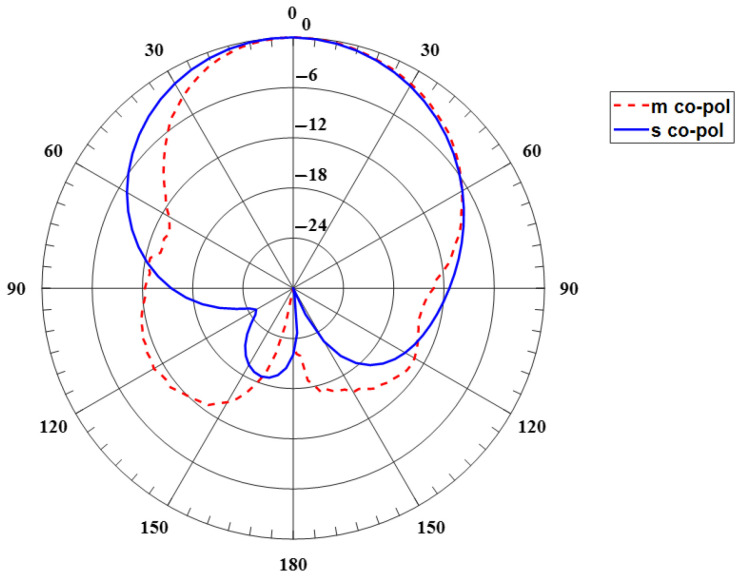
Measured and simulated the radiation pattern of 3D aperture-fed annular ring antenna at 2.4 GHz.

**Figure 10:**
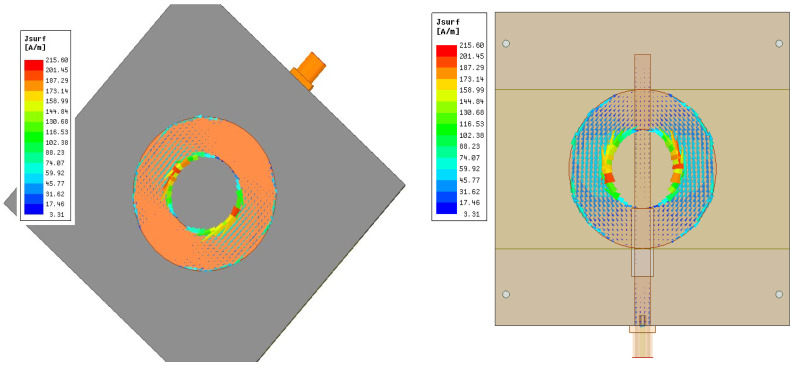
Current distribution of TM11 mode of annular ring antenna.

Based on the previous illustrated design, a 3D-printed antenna with dimensions tabulated in Table 2 was measured. The 3D-optimized antenna is resonant at 2.47 GHz, with a perfect S_11_ (dB) of around 19 dB and good matching with simulated results, as shown in Figure 8. The characteristics of the antennas are tabulated in Table 10. The bandwidth does not change from the probe feeding the annular ring antenna. The radiation pattern is depicted in Figure 9. The overall radiation pattern matches the simulated one. However, the distortion at the left half of the radiation pattern is due to imperfect manufacturing on the substrate and metallic trace. The measured gain is 3.3 dB, around 1.2 dB less than the simulated gain. The efficiency was reduced by around 18% with the measured results. The low infill percentage of the substrate plays a vital role in the high gain as it has a low complex permittivity. The front-to-back ratio level is high as expected, which is 23 dB and 30 dB for simulation and measurement. The optimized antenna, as shown in Figure 10, from the current distribution, operates on TM_11_.

## 5. Conclusions

The design of the AFAR microstrip antenna using the microstrip line feeding is demonstrated. The solderless approach of aperture fed is practical since some 2D materials have a limited capacity to handle high temperatures. Each one of the parametric studies is critical and affects the design. However, the width and length of the aperture are directly related to the excitation and the front-to-back ratio. In particular, these two parameters are a trade-off between good impedance matching and minimizing the back lobe of the radiation patterns. Moreover, the thickness of the radiator substrate is crucial since less is better for maximum power transfer but will cause a lower gain. However, the substrate of the feeding line must be thin to reduce the back lobe. Considering all of these design aspects, an optimized design was implemented using a 3D-printed ABS substrate. The performance was as expected, with suitable impedance matching with a gain of around 3 dB and a front-to-back ratio of about 30 dB. This study shows a promising result and a building block to extend this work further to be applied using a 2D metallic trace material for specific applications.

## Figures and Tables

**Table 1:** Antenna comparison in terms of material, feeding, printing infill, frequency, and gain.

Shape	Material	Feeding	Area cm^2^	Conductor	Infill %	Frequency	Gain
Dipole [47]	ABS	Probe	20	Carbone paste	100	3 GHz	1
Dipole [36]	Nylon	Probe	20	Graphene	100	3 GHz	2.23
Rectangular [3]	NinjaFlex	Probe	-	Copper sheet	100	2.4	−4
Rectangular [48]	NinjaFlex and ABS	Probe	73	Copper sheet	20, 40, and 60	2.3	4, 6 & 5
Bow [23]	PLA	Probe	30	ABS conductive	100	7	-
Inverter [37]	ABS	Probe	25	Silver paste	100	2.4	5.6
Rectangular [50]	PLA	Probe	100	Copper sheet	100	2.4	3
Rectangular [50]	PLA	Probe	100	PLA conductive	100	2.4	1
Annular ring [5]	ABS	Probe	100	Copper sheet	100, 50, 10	2.4, 5.4	5.1, 6.5, 8.0
This work	ABS	Aperture	62	Copper sheet	100 and 50	2.4	3.3

**Table 2:** The antenna aperture feeding design parameters of baseline and 3D ABS.

Dimensions (mm)	Baseline 2.4 GHz	Optimized 3D 2.4 GHz
Annular ring outer radius	20	20
Annular ring inner radius	10	10
Antenna width	75	78
Antenna length	80	80
Patch layer thickness	0.1	1.52
Feeding layer thickness	0.5	1.52
Substrate of feed line	RT4003	ABS 100 infill
Complex permittivity	3.55–0.009i	2.5–0.12i
Substrate of radiator	PET	ABS 3D 50 infill
Complex permittivity	2.8–0.008i	2.2–0.09i

**Table 3:** A comparison of the baseline performance of different feeding line thickness substrates with the same radiator’s substrate thickness of 60 mils.

Feeding’s Substrate Thickness (mm)	L_Apt_	W_Apt_	Frequency (GHz)	RL (dB)	Gain (dB)	Front-to-Back Ratio
0.5	7	12	2.45	15	5.2	13
1.52			2.46	7	4.9	20
0.5	17	6	2.44	5	4.7	13
1.52			2.46	15	4.3	22

**Table 4:** The performance of different L_Stub_ lengths.

(L_Stub_)	Frequency (GHz)	RL (dB)	$Z_{in(Real)}$	$Z_{in(Img.)}$
10	2.46	11	87	28
20	2.49	9.8	27	−12
30	2.47	6	1.5	15
40	2.47	0.8	3	30
48	2.47	6.5	34	47
50	2.46	8.9	65	41
60	2.49	15	39	10

**Table 5:** The performance of different aperture widths (W_Apt_) with fixed lengths (7 mm).

W_Apt_	Frequency (GHz)	RL (dB)	Gain (dB)	Front-to-Back Ratio
4	2.35	1.5	3	18
6	2.45	3	4	19
8	2.46	4	4.6	21.05
10	2.46	6	4.8	21
12	2.46	7	4.9	19.9

**Table 6:** The performance of different aperture lengths (L_Apt_) with fixed aperture width (W_Apt_) (12 mm).

L_Apt_	Frequency (GHz)	RL (dB)
14	2.35	1.1
16	2.39	1.5
18	2.37	2
20	2.39	3
22	2.41	4
24	2.42	4.5
26	2.44	5
28	2.48	4.5
30	2.5	4.2

**Table 7:** The performance of different aperture lengths (L_Apt_) with aperture width (W_Apt_) (6 mm).

L_Apt_	Frequency (GHz)	RL (dB)	Gain (dB)	Front-to-Back Ratio
14	2.47	12	5	21
16	2.47	17	5	20.4
17	2.47	20	5	23
18	2.47	22	5	20.3
20	2.47	36	5	20
22	2.47	25	5	20.7
24	2.48	28	5	20.1
26	2.48	23	5	19
28	2.48	18	5	18
30	2.5	17	5	18

**Table 8:** The performance of different aperture widths (W_Apt_) with fixed length (L_Apt_) (17 mm).

W_Apt_	Frequency (GHz)	RL (dB)	$Z_{in(Real)}$	$Z_{in(Img.)}$	Front-to-Back Ratio	Gain (dB)
4	2.45	4	41	65	4.3	20.9
6	2.47	20	70	12	5	23
8	2.47	13	50	−27	5	22
10	2.47	7.4	35	−39	4.9	21
12	2.45	6	53	63	4.5	23.3

**Table 9:** 3D-optimized aperture feeding annular ring antenna dimensions with respect to the feeding line end connector.

AFAR 2.4 GHz	$\lambda_{g}(\mathrm{mm})$	Optimized 3D 2.4 GHz (mm)
L_feed_	0.25	19.5
L_Stub_	0.62	48.5
L_Apt_	0.21	16.5
W_Apt_	0.07	5.5

**Table 10:** Optimized aperture feeding annular ring antenna characteristics (resonance frequency, return loss, gain, axial ratio, and polarization).

	Aperture Feeding	
ARA Microstrip	Sim.	Meas.
F (GHz)	2.47	2.47
RL (dB)	17	19
Bandwidth (MHz)	30	29
Bandwidth %	1.21	1.20
Gain (dBi)	4.6	3.3
Efficiency %	60	42
Back lobe	23	30
Axial ratio (dB)	55	>20
Polarization	Linearly	Linearly

## Data Availability

Data is available upon request.
